# Integrated multi-omics landscape of non-small cell lung cancer with distant metastasis

**DOI:** 10.3389/fimmu.2025.1560724

**Published:** 2025-03-17

**Authors:** Teng He, Ting Jiang, Xiaoyuan Sun, Fang Yang, Dan Zhang, Shan Yao, Jiangrong Liao, Xueling Wu

**Affiliations:** ^1^ Department of Respiratory and Critical Care Medicine, Ren Ji Hospital, Shanghai Jiao Tong University School of Medicine, Shanghai, China; ^2^ Department of Respiratory and Critical Care Medicine, Guizhou Aerospace Hospital, Guizhou, China

**Keywords:** lung cancer, metastasis, exosome, proteomics, metabolomic

## Abstract

**Background:**

Distant metastasis is one of the important factors affecting the prognosis of lung cancer patients. Extracellular vesicles (EVs) play an important role in the occurrence, development, and metastasis of cancer. However, it is currently unclear whether EVs in BALF are involved in distant tumor metastasis.

**Methods:**

we collected bronchoalveolar lavage fluid (BALF) from patients with metastatic and non-metastatic non-small cell lung cancer (NSCLC) to isolate exosomes, which were then characterized by nanoparticle tracking analysis (NTA) and transmission electron microscopy (TEM), followed by comprehensive metabolomic and proteomic analysis to ultimately construct a distant metastasis prediction model for non-small cell lung cancer.

**Results:**

Our research has found that the BALF of NSCLC patients is rich in EVs, which have typical morphology and size. There are significant differences in protein expression and metabolite types between patients with distant metastasis and those without distant metastasis. Sphingolipid metabolism pathways may be a key factor influencing distant metastasis in NSCLC. Subsequently, we constructed a predictive model for distant metastasis in NSCLC based on differentially expressed proteins identified by proteomics. This model has been proven to have high predictive value.

**Conclusion:**

The multi-omic analysis generated in this study provided a global overview of the molecular changes, which may provide useful insight into the therapy and prognosis of NSCLC metastasis

## Introduction

Regardless of the great advances made in lung cancer treatment such as molecular targeted therapies and immunologic control over the past decade, it remains one of the most deadliest forms of cancer globally, accounting for 24% of all cancer deaths (1). Approximately 57% of lung cancer patients have already developed distant metastasis at the time of initial diagnosis. Furthermore, tumor metastasis accounts for 67%-90% of all cancer mortality, with the figure exceeding 70% in lung cancer (2). Brain metastasis is one of the most common metastatic sites. The incidence rate is about 7.3 cases per 100000 people (3), of which about half are NSCLC patients. Furthermore, the one-year survival rate of NSCLC patients with distant metastasis is only 15% -19% (4). Therefore, the study of mechanisms of lung cancer metastasis will be of great significance.

Exosomes are a subtype of EVs, which are lipid bilayer-enclosed vesicles, spherical or cup-shaped, with diameters ranging from 30 to 150nm (5). Whether under physiological or pathological conditions, almost all cells secrete exosomes, widely distributed in physiological fluids, including blood, saliva, urine, and bronchoalveolar lavage fluid (6). They play a critical role in cell-cell communication and can regulate various biological and pathological processes such as inflammation and immune responses, tumor growth, and metastasis (7).

Lung tumor-derived exosomes has received increasing research attention in metastatic activity integrated with immune system evasion, epithelia-mesenchymal transition and angiogenesis. It has been demonstrated that tumor exosome integrins (ITGs) determine organotropic metastasis with exosome proteomics. Exosome-based ITGα6β4 and ITGα6β1 were associated with lung metastasis, while ITGαvβ5 was linked to liver metastasis. In their observations, exosome integrin isolated from circulating plasma of cancer patients uptake by resident cells activates Src phosphorylation and pro-inflammatory S100 gene expression (8). A recent study with plasma exosomes from lung cancer cases having liver metastasis (LM) or bone metastasis (BM) was subjected to proteomic profiles. SELL and MUC5B could be used as diagnostic markers of BM, while APOH, CD81, and CCT5 could help diagnose LM in local advanced lung cancer patients (9). Hypoxic bone marrow-derived mesenchymal stem cells (BMSC)-derived exosomal miRNAs promote metastasis of lung cancer with a microRNA array (10). Further study demonstrated that plasma exosome-mediated transfer of miR-193a-3p, miR-210-3p and miR-5100 could promote invasion of lung cancer cells by activating STAT3 signaling-induced EMT. Using lncRNA-seq, a novel exosomal long noncoding RNA (lncRNA), lnc-MLETA1 was found to promote tumor metastasis by regulating the miR-186-5p/EGFR and miR-497-5p/IGF1R axes in NSCLC (11).

BALF has been considered to be a sample that best represents the condition of pulmonary diseases, except for lung biopsy and surgical specimen. Exosomes in BALF has been shown to be a key factor involved in the growth and progression of lung cancer *in vitro* and *in vivo* (12). Certainly, BALF also has limitations, such as low protein content, the need for invasive procedures to obtain it, and the risk of contamination. However, knowledge of the role of BALF exosomes highlighting for being as biomarkers in diagnosis of cancer metastasis remain acquaint scarcely. In this study, exosomes extracted from BALF of patients with NSCLC were confirmed through NTA. Proteomic and metabolic analyses were subsequently conducted to explore the role of exosomes in NSCLC.

## Methods

### Ethical approval and clinical specimen collection

The study involving human participants was reviewed and approved by the Renji Hospital, Shanghai Jiaotong University School of Medicine, China (No. LY2024-179-A) and Guizhou Aerospace Hospital, China (No. (2024)1-050), and providing written informed consent in accordance with the regulations of the relevant Hospital Organizational Review Board and the Declaration of Helsinki. NSCLC patients were collected from June 2023 to June 2024 at the respiratory department of the aforementioned hospital. All patients were over 18 years old, confirmed by pathology as NSCLC with or without distant metastasis, and were able to obtain complete and comprehensive clinical data. Exclude patients with infectious diseases such as active tuberculosis, HIV, and viral hepatitis, other types of tumors, and those who refuse or are unable to cooperate with bronchial examinations. All patients received preliminary diagnosis and obtained BALF from the aforementioned hospital.

### EV purification and characterization

BALF-derived EVs were isolated with a combined chromatography kit, following the manufacturer’s instructions. Briefly, 1 ml of BALF was concentrated using a 30K molecular weight cut-off (MWCO) spin filter (Amicon, Merck Millipore, MA, USA). Then 100 μL of concentrated BALF were loaded onto columns pre-packed with 500μL of Sepharose CL-6B resins (Cytiva). 200 μL filtrate were collected and added to columns containing a specific mixture of Fractogel EMD SO3- (M) (Millipore Sigma) and Capto Core 700 (Cytiva). After incubation, the columns were centrifuged at 500 x g for 30 seconds to separate and isolate the EVs, which were then prepared for further downstream analysis.

EVs were characterized by NTA and TEM. NTA was carried out using a ZetaView instrument (Particle Metrix, Germany) to assess the particle size distribution and concentration. For NTA measurement, according to the ZetaView manual, routine calibration and instrument performance verification were performed using NanoStandard™ Series 100 nm standards. Before each sample injection, the instrument was flushed with PBS to ensure the absence of interfering particles within the lens. Samples were diluted with PBS to achieve 50-400 particles per frame. Subsequently, three cycles were performed by scanning 11 cell positions each and capturing 30 frames per position.

TEM was utilized to examine the morphological features of the EVs. A 30 μl aliquot of the sample suspension was placed on a carbon-coated copper grid for 10 minutes. Excess liquid was blotted away, followed by staining with uranyl acetate solution. After removing the residual staining solution, the grid was air-dried on filter paper for 3 hours. Subsequently, the samples were then observed under the microscope (JEOL, Japan).

### Proteomics sequencing

All samples were analyzed for proteomics by EVbio, China. Extract proteins from the above samples using protease inhibitors and ultrasonic lysis. Subsequently, reduction alkylation, SP3 enzyme digestion, and desalination treatment were performed, and the Vanquish Neo/Astral mass spectrometer was used to generate mass spectrometry detection raw data in data-independent acquisition (DIA) mode. The raw MS files were analyzed and searched against target protein database based on the species of the samples using DIA-NN. The parameters were set as follows: the protein modifications were carbamidomethylation (C) (fixed), oxidation (M) (variable), Acetyl (Protein N-term) (variable), the enzyme specificity was set to trypsin, the maximum missed cleavages were set to 2, Precursor Qvalue Cutoff was set to 20 ppm, and Protein Qvalue Cutoff was set to 20ppm.

### Metabolomics sequencing

All samples were analyzed for metabolomics by EVbio, China. Analyze exosome samples using the Thermo Fisher Scientific U3000 liquid chromatograph, and perform substance annotation identification with Progenesis QI software. After peak alignment, baseline correction, and noise reduction, proceed with feature alignment as well as quantitative and qualitative analysis. Principles for quantitative screening of metabolites: RSD value is less than 30, more than 50% of the QC samples have expression (including 50%), and more than 30% of the experimental samples have expression (including 30%). Principles for qualitative screening of metabolites: eliminate metabolites with a fragmentation score of less than 30, and deduplicate the annotation results from the HMDB database.

### Construction of a biomarker prediction model

We have constructed a predictive model for distant metastasis of NSCLC using selected proteins. This model is based on the Least Absolute Shrinkage and Selection Operator (LASSO) analysis, modeled with neural networks, and finally evaluated using the receiver operating characteristic (ROC) curve with an area under the curve (AUC) to calculate the diagnostic performance of the model.

### Statistical analysis

Mann-Whitney U tests were used for comparisons of continuous variables, whereas Chi-square tests were used for categorical variables. Correlation analysis was assessed by Spearman correlation. P value of < 0.05 (two-tailed) was considered statistically significant.

## Results

### Participants and exosome characteristics

**Figure 1:**
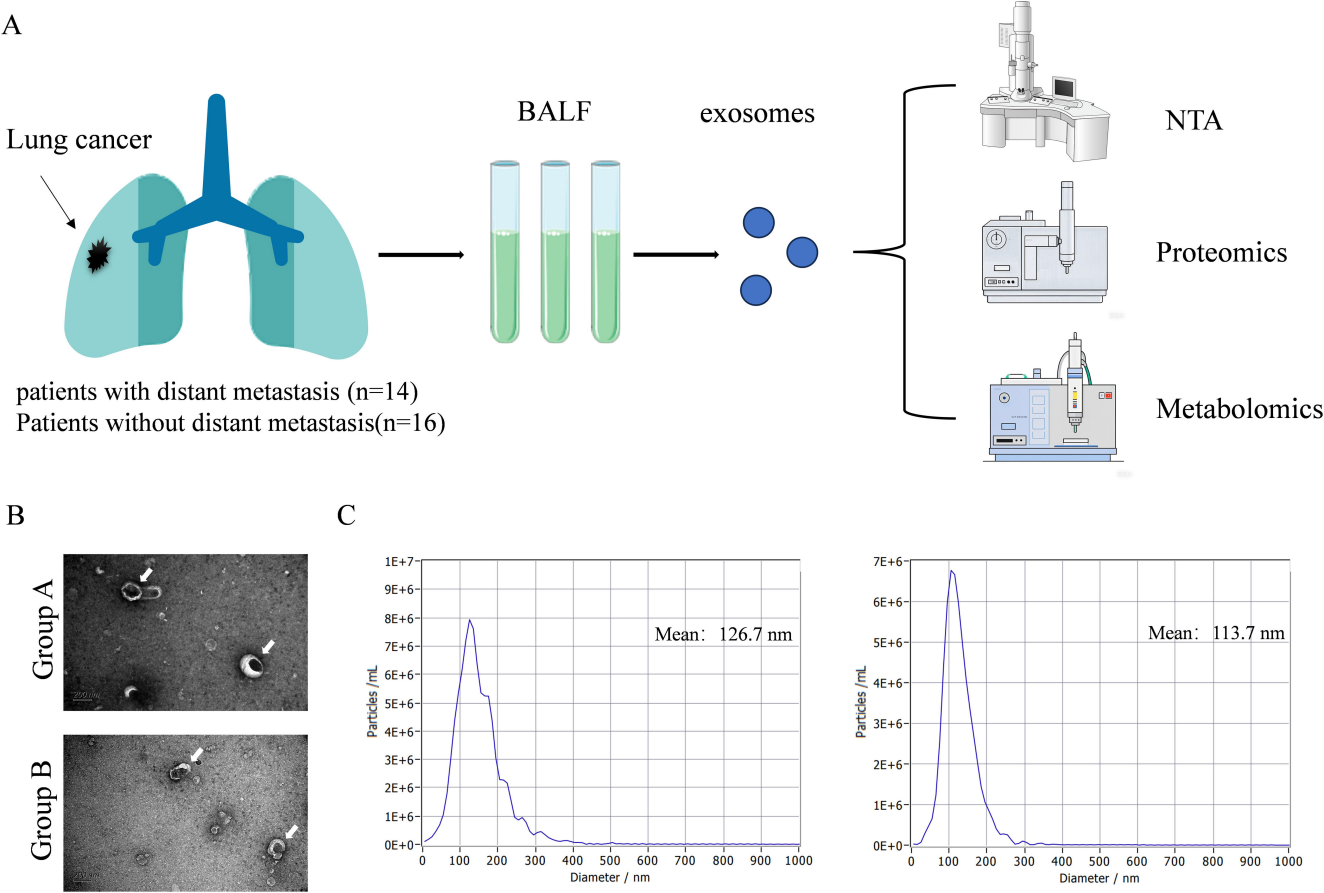
Overview of research design and characteristics of EVs. **(A)** The workflow of proteomics and omics experiments for BALF samples. **(B)** Typical TEM images of EVs in BALF (white arrows). Scale bar: 200 nm. **(C)** Using NAT to obtain typical images of the particle size distribution and concentration of EVs in BALF.

We collected BALF samples from 30 NSCLC patients, including 14 patients with distant metastasis (Group A) and 16 patients with non-distant metastasis (Group B). The clinical characteristics of patient are shown in **Table 1**. The workflow of proteomics and omics experiments for BALF samples is shown in **Figure 1A**. The average age of Group A is 67.6 (SD, 9.2) with males accounting for 78.6%. The average age of Group B is 68.8 (SD, 9.5) with males accounting for 81.3%. There was no significant difference in laboratory tests between Group A and Group B (**Table 2**). We conducted an analysis of the collected patient BALF using TEM and NTA identified exosomes with typical morphological and size characteristics (**Figure 1B**), with an average diameter of 126.7 nm in Group A and 113.7 nm in Group B (**Figure 1C**).

**Table 1:** Basic characteristics of patients.

Characteristic	Group *A* (n = 14)	Group B (n = 16)	P value
Age (year), mean ± SD	67.6 ± 9.2	68.8 ± 9.5	0.560
Sex, no. (%)			0.857
Male	11 (78.6)	13 (813)	
Female	3 (21.4)	3 (18.7)	
Tumor site no. (%)			0.154
Left	9 (64.3)	6 (37.5)	
Right	5 (35.7)	10 (62.5)	
Histological type, no. (%)			0.382
Adenocarcinoma	7 (50)	7 (43.8)	
squamous carcmoma	7 (50)	9 (56.2)	
Smoking status, no. (%)			0.659
Never smoker	9 (64.3)	9 (56.3)	
Ex-smoker	5 (35.7)	7 (43.7)	
Hypertension, no. (%)			0.951
Yes	5 (35.7)	6 (37.5)	
No	9 (64.3)	10 (62.5)	
Diabetes, no. (%)			0.608
Yes	0 (0)	3 (18 8)	
No	14 (100)	13 (81.2)	
Chronic obstructive pulmonary disease, no. (%)			0.275
Yes	6 (42.9)	3 (18.8)	
No	8 (57.1)	13 (81.2)	
Clinical T stage, no. (%)			0.12
Tl	0 (20)	2 (12.5)	
T2	4 (28.6)	6 (37.5)	
T3	2 (14.3)	3 (18.7)	
T4	8 (57.1)	5 (313)	
Clinical N stage, no. (%)			0.113
NO	4 (28.6)	9 (56.25)	
Nl	1 (7.1)	1 (6.25)	
N2	5 (35.7)	4 (25)	
N3	4 (28.6)	2 (12.5)	
Clinical stage group, no. (%)			< 0.001***
I	0 (0)	2 (12.5)	
II	0 (0)	5 (31.2)	
III	0 (0)	9 (56.3)	
IV	14 (100)	0 (0)	

(***P < 0.001).

**Table 2:** Hematological examination characteristics of patients.

Characteristic	Group *A* (n = 14)	Group B (n = 16)	P value
WBC (*109/L)	7.4 (5.64-10.49)	6.9 (4.6-11.29)	0.561
NEU (*109/L)	5.4 (3.42-8.59)	4.9 (2.72-8.67)	0.244
LYM (*l 09/L)	1.7 (0.65-2.11)	1.3 (0.81-1.86)	0.708
Hb (g/L)	125 (91-164)	116 (71-143)	0.56
PLT (*l 09/L)	254.2 (147-387)	293.1 (191-469)	0.318
CRP (mg/L)	52.3 (0.5-117.06)	33.6 (0.5-146.58 )	0.088
APTT (second)	28.5 (23 1-33.4)	28.5 (25 9-31.2)	0.934
ALT (U/L)	18.5 (8-33)	17.4 (7-26)	0.803
AST (U/L)	23.5 (14.01-38)	23.3 (13-28.37)	0.983
STB (µmol/L)	12.8 (5.1-46.07)	23.5 (14.01-38)	0.835
CB (µmo l/L)	2.8 (1-9.05)	5.7 (1-11.45)	0.934
BUN (mmol/L)	5.3 (2.55-10.2)	9.2 (3.29-62)	0.589
Cr (µmol/L)	74.5 (37. 14-124.5)	76 (53-125.4)	0.868

### Proteomic changes and biological pathways of EVs in BALF of NSCLC patients

**Figure 2:**
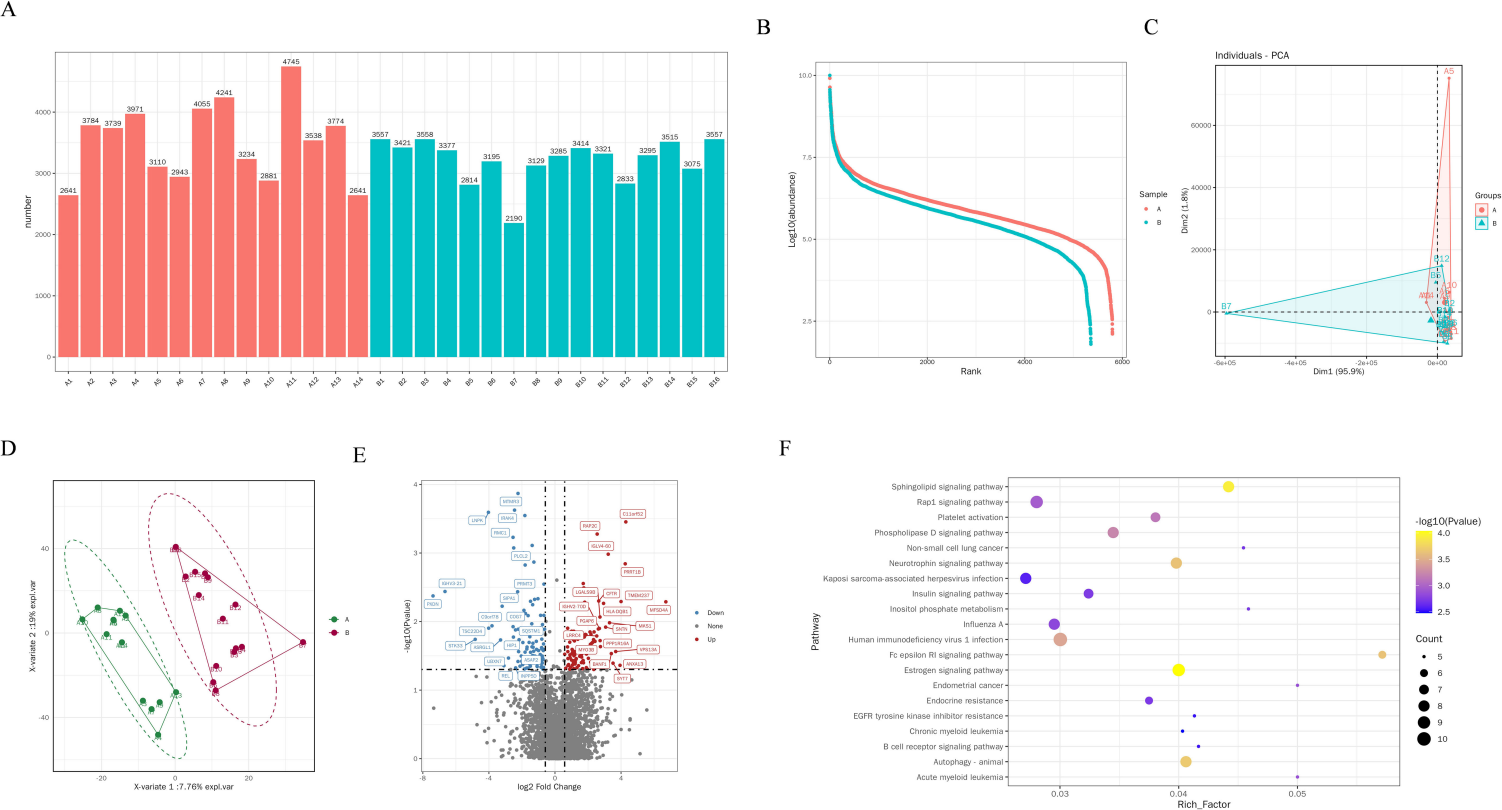
Proteomic changes and biological pathways of EVs in BALF of NSCLC patients. **(A)** Quantity of EVs proteins in BALF of each patient. **(B)** Differences in protein abundance between two groups of EVs. **(C)** PCA analysis of EVs proteins in distant metastasis group and non-distant metastasis group of lung cancer. **(D)** PLS-DA analysis of EVs proteins in group A and group B **(E)** Volcano plot shows the differential proteins between the group A and group B, each dot represents a protein, with red dots for proteins significantly upregulated in brain metastasis group and blue dots for proteins significantly downregulated in brain metastasis group. Fold change ≥ 2.0 and P < 0.05 is considered statistically significant. **(F)** KEGG pathway analysis of differential proteins.

After filtering the low abundant proteins, 4255 high-quality proteins were collected for the data analysis. The median protein number for 30 samples was 3361 (**Figures 2A, B**). Our preliminary analysis employed PCA (**Figure 2C**) and PLS-DA (**Figure 2D**) method to explore the clustering patterns among the groups, which demonstrated a distinct separation between Group A and Group B. A comprehensive differential expression analysis revealed that 209 proteins exhibited unique changes in NSCLC patients with distant metastasis compared to those without, with 109 proteins showing continuous downregulation and 100 proteins showing upregulation (**Figure 2E**). These findings demonstrated the characteristics of serum protein alterations correlating with the metastasis of the disease. Among the upregulated proteins, Myotubularin-related protein 3 (MTMR3) (**Supplementary Figure S1A**) and Interleukin-1 Receptor Associated Kinase 1(IRAK4) (**Supplementary Figure S1B**) are the two most significantly altered proteins. On the contrary, transmembrane conductance regulator (CFTR) is one of the most significantly downregulated proteins (**Supplementary Figure S1C**). Subsequently, KEGG analysis showed significant changes in the following pathways, Human immunodeficiency virus 1 infection, Autophagy, Rap1 signaling pathway, Estrogen signaling pathway, Sphingolipid signaling pathway, Phospholipase D signaling pathway, Neurotrophin signaling pathway (**Figure 2F**).

### Metabolomic changes of EVs in BALF of lung cancer patients

**Figure 3:**
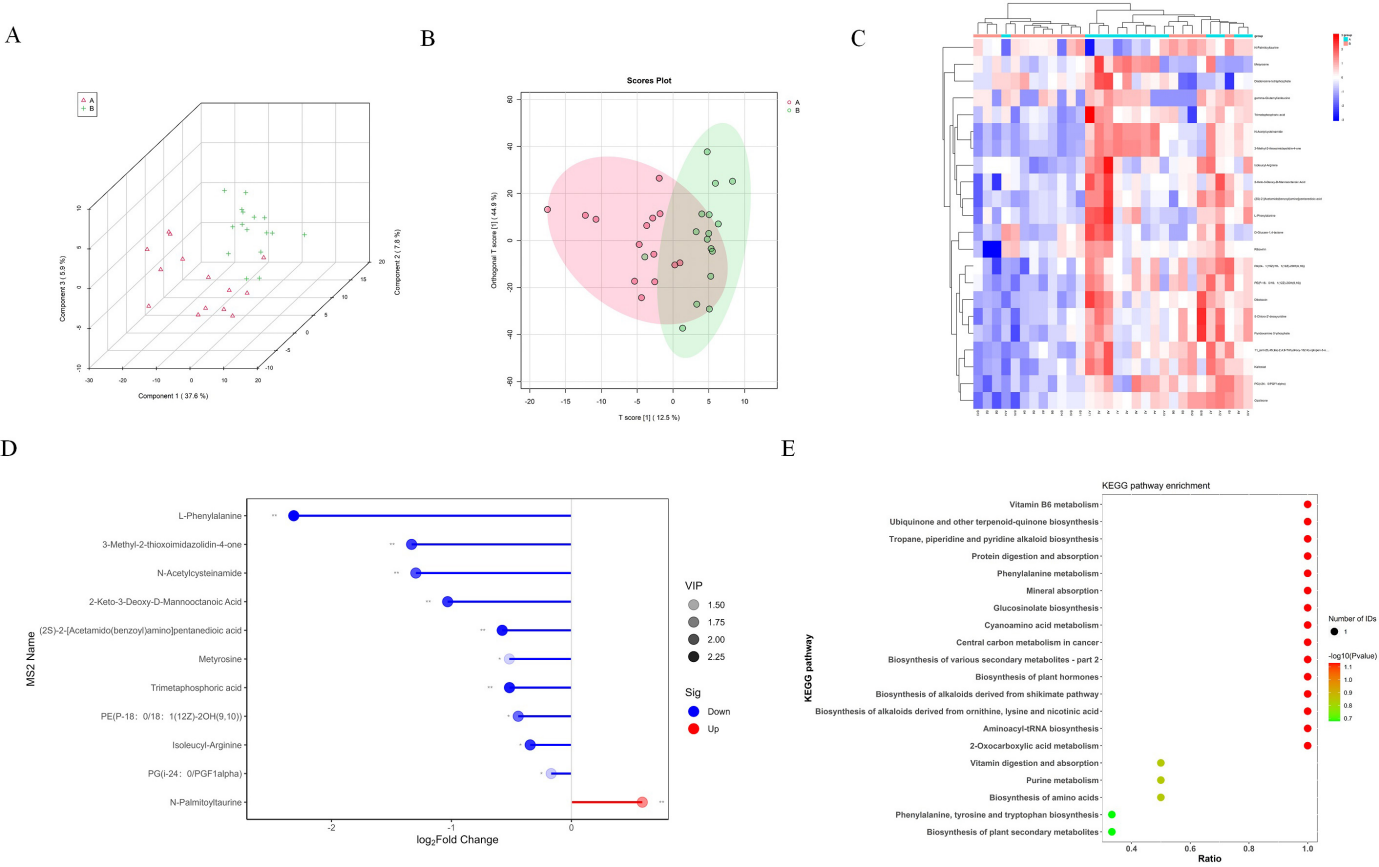
Metabolomic changes of EVs in BALF of NSCLC patients. **(A)** PLS analysis of EVs metabolites in group A and group B **(B)** OPLS analysis of EVs metabolites in group A and group B **(C)** The heatmap showed the 22 metabolites with significant changes. **(D)** The lollipop chart shows the 10 down regulated proteins and 1 up regulated protein with the most significant changes in the distant metastasis group of lung cancer. **(E)** KEGG pathway analysis of differential metabolites.

In our metabolic investigation, we cataloged a comprehensive array of 651 metabolites, encompassing amino acids, lipids, and other critical metabolites. The PLS and OPLS plots revealed a pronounced metabolic differentiation in B VS A groups (**Figures 3A, B**). The most significant changes in metabolites include 2-Keto-3-Deoxy-D-Manooctanoic Acid,3-Methyl-2-thioxoimidazolidin-4-one, L-Phenylalanine, Metyrosine, N-Palmitoyltaurine, PE(P-18:0_18:1(12Z)-2OH(9,10)), PG(i-24:0_PGF1alpha), Isoleucyl-Arginine, N-Acetylcysteinamide, Trimetaphosphoric acid (**Figures 3C, D**). Next, we further analyzed the signaling pathways involved through KEGG analysis, including Biosynthesis of alkaloids derived from, Aminoacyl-tRNA biosynthesis, Protein digestion and absorption, Biosynthesis of plant hormones, Biosynthesis of various secondary, 2-Oxocarboxylic acid metabolism, Phenylalanine metabolism, Central carbon metabolism in cancer, Cyanoamino acid metabolism, Mineral absorption, Glucosinolate biosynthesis, Biosynthesis of amino acids, Biosynthesis of plant secondary metabolites, Phenylalanine, tyrosine and tryptophan biosynthesis, Biosynthesis of phenylpropanoids, Phenylpropanoid biosynthesis, Biosynthesis of antibiotics, ABC transporters (**Figure 3E**).

### Biomarker panel for early prediction of distant metastasis in NSCLC patients

**Figure 4:**
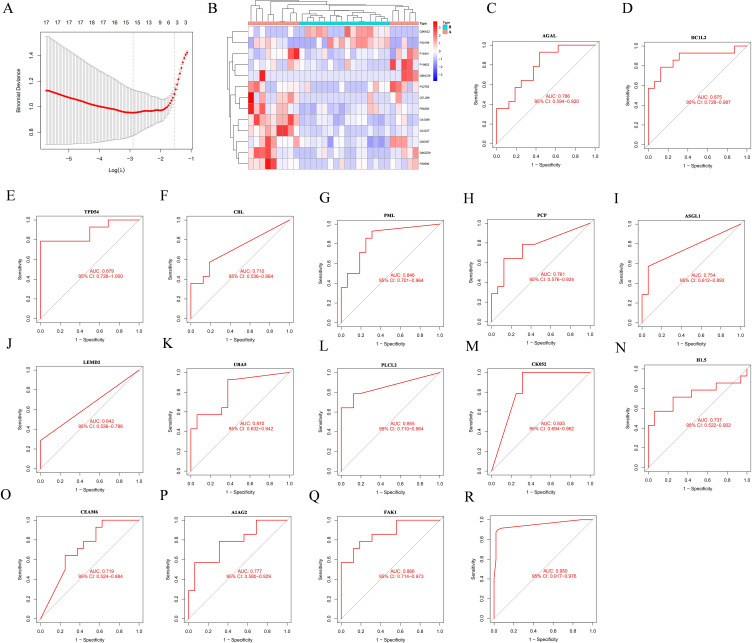
Biomarker panel for early prediction of distant metastasis in NSCLC patients. **(A)** Constructing LASSO regression analysis model using differential proteins. **(B)** Prediction model of biomarkers for distant metastasis of lung cancer containing 15 proteins. **(C–Q)** Receiver operating characteristic curve (ROC) of AGAL, DCIL2, TPD54, CBL, PML, PCP, ASGL1, LEMD2, UBA5, PLCL2, CK052, H1.5, CEAM6, A1AG2 and FAK1. **(R)** The overall predictive performance of protein models. 95% confidence interval displayed on the graph.

We have constructed a biomarker model for the early prediction of lung cancer distant metastasis using the aforementioned proteomics data. 662 candidate variables obtained proteomics were screened by LASSO regression analysis (**Figure 4A**). Ultimately, a prediction model for lung cancer distant metastasis composed of 15 proteins was obtained (**Figure 4B**). Subsequently, we analyzed the Area Under the Curve (AUC) for each of the 15 proteins including AGAL, DCIL2, TPD54, CBL, PML, PCP, ASGL1, LEMD2, UBA5, PLCL2, CK052, H1.5, CEAM6, A1AG2 and FAK1 and found that their diagnostic performance ranged from 0.648 to 0.879 (**Figures 4C–Q**). The protein model composed of 15 types of proteins has shown excellent predictive power, with an AUC of 0.95 (95% CI 0.917-0.976), which proves this point (**Figure 4R**).

## Discussion

This study is the first to use exosomes from patients’ BALF as the research object, and systematically elucidates the differences between NSCLC patients with metastasis and those without metastasis by combining proteomics and metabolomics. Furthermore, we utilized the aforementioned results to construct a predictive model containing 15 proteins, providing reliable biomarkers for the prediction of distant metastasis in NSCLC patients.

BALF is a common method of injecting saline into the alveoli and then retrieving alveolar cell components and secretions through negative pressure aspiration, which can well reflect the changes within the lung tissue and is therefore referred to as a liquid biopsy (13, 14). TME is shaped and conditioned by cancer cells, and it also serves as a critical target for therapy (15). EVs in BALF have become a new means of intercellular communication (16). Similarly, our research found that BALF of NSCLC patients contains a rich array of exosomes, which was confirmed by NTA.

Subsequently, we performed proteomic analysis on the collected exosome samples. The results indicated that there were abundant differences in protein expression between the distant metastasis group and the non-distant metastasis group. Moreover, KEGG analysis suggested the potential role of the sphingolipid signaling pathway particularly the key protein MTMR3. MTMR3 is a member of the myotubularin protein family (17). Essentially, it is a phosphatidylinositol 3-phosphatase that can hydrolyze phosphatidylinositol-3-phosphate, affecting phosphatidylinositol metabolism and thus participating in the sphingolipid metabolism pathway. The MTMR3 protein has been confirmed to be associated with the proliferation, migration, and autophagy of tumor cells in breast cancer (18, 19). Additionally, the MTMR3 protein plays an important role in phosphoinositide metabolism and is a crucial component of the sphingolipid metabolic pathway (17). Research indicates that the high expression level of MTMR3 may be correlated with a lower overall survival period and a shorter recurrence-free survival period in breast cancer patients (19). The underlying mechanism might be caused by MTMR3 influencing the transition of tumor cells from G1 to S phase. In oral cancer, miR-99a can inhibit the proliferation, migration and invasion of tumor cells by reducing the expression of MTMR3 (20). IRAK-4-NF-kB is an important component of the Toll-like receptor (TLR) signaling pathway (21). Immunohistochemical studies on melanoma have shown that phosphorylated IRAK-4 is widely expressed in diseased tissues (22). Moreover, silencing the gene expression of IRAK-4 by shRNA or the application of small molecule inhibitors can induce the proliferation of tumor cells in patients with T-acute lymphoblastic leukemia, suggesting that the signaling mediated by IRAK-4 is an important factor in disease progression (23). Interestingly, the inflammatory response can promote the expression of various inflammatory factors by activating IRAK-4- NF-kB, thereby participating in sphingolipid metabolism (24).

Furthermore, metabolomics analysis indicates that multiple significantly different metabolites are associated with the sphingolipid signaling pathway. Phosphatidylethanolamine (PE) itself is the final product of sphingolipid degradation. Specifically, sphingosine 1-phosphate (S1P) is decomposed into PE and hexadecenal under the action of sphingosine-1-phosphate lyase (SPL) (25). Additionally, PE serves as a primary supplier of phosphate groups in the synthesis of sphingomyelin (26). N-Palmitoyltaurine is a derivative of taurine, which is an important component of sphingosine in the brain. In addition, L-Phenylalanine is one of the precursors for the synthesis of ceramide, and it can also serve as a substrate to participate in the synthesis of sphingosine, thereby affecting sphingolipid metabolism. Research indicates that a novel probiotic, Christensenella minuta, can regulate cytochrome P450 metabolism, sphingolipid metabolism, PI3K-AKT pathway, and integrin pathway by affecting phenylalanine metabolism, thereby alleviating acetaminophen mediated liver injury (27). All of these evidences support the potential role of sphingolipid metabolism in the distant metastasis of NSCLC.

In this study, based on the results of exosomal proteomics, we constructed a model containing 15 proteins for predicting distant metastasis in NSCLC patients. This model provides an example for early identification of distant metastases in NSCLC. Interestingly, TPD54 has been shown to interact with intracellular nanovesicles and play a role in regulating cell migration and invasion. A previous study revealed that in prostate cancer patients, the mRNA and protein expression levels of TPD54 were significantly upregulated and closely associated with poor prognosis (28). *In vitro* experiments demonstrated that overexpression of the TPD54 gene in oral squamous cell carcinoma cells significantly increased the size of cell colonies (29). The Casitas B-lineage lymphoma (CBL) family, as a class of ubiquitin ligases comprising c-CBL, CBL-b, and CBL-c, can regulate signal transduction in multiple tyrosine kinase-dependent pathways to mediate tumor growth (30). Studies have shown that Cbl-b is upregulated in exhausted (PD1+Tim3+) CD8+ T cells, and knocking out the Cbl-b gene in CAR-T cells renders them resistant to exhaustion (31). Furthermore, in NSCLC xenograft models, reducing CBL-c expression diminishes tumor cell viability and clonogenicity, mediating the inhibition of tumor growth. Interestingly, silencing CBL-c expression significantly enhances the sensitivity of EGFR-mutant NSCLC cells to tyrosine kinase inhibitors. Therefore, targeting CBL-c represents a promising strategy to suppress tumor growth and improve the therapeutic response of EGFR-mutant NSCLC to tyrosine kinase inhibitor treatment (32). Similarly, by reducing the autophosphorylation kinase activity of focal adhesion kinase 1 (FAK1), the cell proliferation of FC-IBC02, SUM190, and KPL4 can be decreased (33). In summary, these biomarkers are closely related to the proliferation, migration, and invasion of tumor cells. In summary, the expression of these 15 proteins is closely associated with tumor cell proliferation, migration, and invasion. This model achieved an AUC of 0.95 in predicting distant metastasis of NSCLC. Additionally, cancer cells undergo profound alterations in energy metabolism during metastasis. Our study revealed significant changes in the sphingolipid metabolic pathway in NSCLC patients with distant metastasis. Therefore, combining proteomic models with metabolite detection may be one of the approaches to further improve the accuracy of this predictive model. Interestingly, in our study, the non-distant metastasis group included patients with stages I, II, and III, who also have the potential to develop distant metastasis, and there are differences among these stages. Therefore, the heterogeneity in staging between the groups may introduce confounding factors that could influence the results. Consequently, further subgroup analyses are needed to validate the independent effects of different stages versus metastasis itself.

Our research still has limitations. Firstly, further *in vivo* experiments are needed to confirm the role of the sphingolipid metabolism pathway in distant metastasis of NSCLC. Secondly, the distant metastasis prediction model contains a large number of proteins, which limits its widespread clinical application. Finally, the combined analysis of proteomics and metabolomics, along with errors in data processing, may affect the accuracy of the results.

In conclusion, this comprehensive study highlights the importance of lipid metabolism, in elucidating the pathogenesis of NSCLC metastasis. These findings hold significant implications for potential risk assessment and therapeutic strategies in lung cancer. Furthermore, following further detailed validation, the results of this multi-omics analysis are expected to advance toward broader clinical applications.

## Data Availability

The sequencing data for this article has been uploaded to the iProX database with the Project ID: IPX0011284000, and the specific access link is: https://www.iprox.cn/page/project.html?id=IPX0011284000.
