# Supplementary material for: Integrated multi-omics landscape of non-small cell lung cancer with distant metastasis
Source: Front Immunol. 2025 Mar 17;16:1560724. doi: 10.3389/fimmu.2025.1560724 (PMC11956740; doi:10.3389/fimmu.2025.1560724)
Supplement: Supplementary file 1 [file DataSheet1.docx]

**Integrated Multi-Omics Landscape of** **lung cancer with distant metastasis**

Teng He^1, #^, Ting Jiang^2, #^, Xiaoyuan Sun^1, #^, Fang Yang^2, #^, Dan Zhang^2^, Shan Yao^2^, Jiangrong Liao^2, *^, Xueling Wu^1, 3, *^

^1^Department of Respiratory and Critical Care Medicine, Ren Ji Hospital, Shanghai Jiao Tong University School of Medicine, Shanghai, China

^2^Department of Respiratory and Critical Care Medicine, Guizhou Aerospace Hospital, Guizhou, China

^#^These authors contributed equally to this work

^3^Leading Contact: Xueling Wu.

*Correspondence: Jiangrong Liao, [Ljr3409@sina.com](mailto:Ljr3409@sina.com) and Xueling Wu, [wuxueling76@126.com](mailto:wuxueling76@126.com)

**Fig S1**


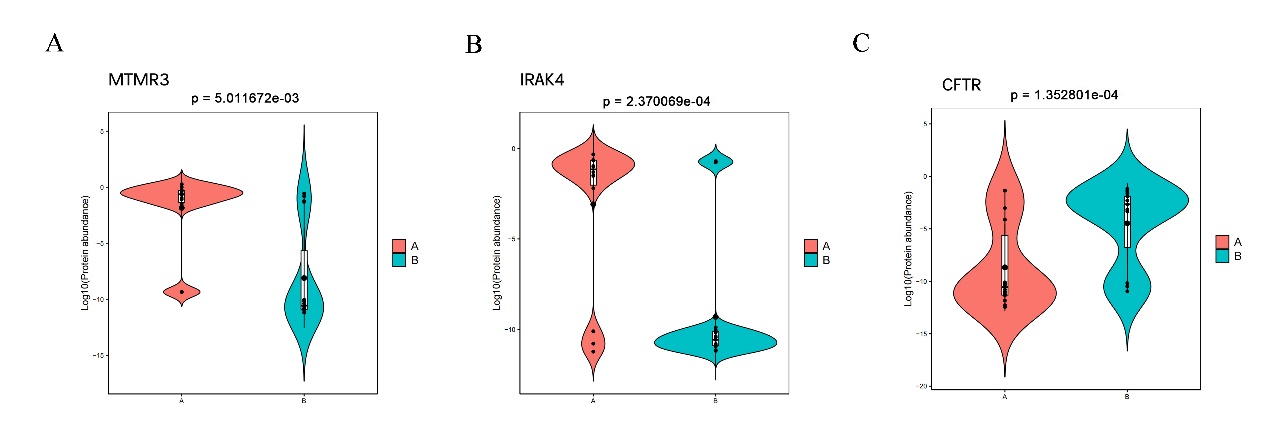


**Figure S1: Differential proteins in extracellular vesicles between distant metastasis group（group A） and non-distant metastasis group of NSCLC（group B）.** (A) MTMR3, (B) IRAK4, (C) CFTR.
